# Efficacy and safety of anticoagulant for treatment and prophylaxis of VTE patients with renal insufficiency: a systemic review and meta-analysis

**DOI:** 10.1186/s12959-023-00576-2

**Published:** 2024-02-05

**Authors:** Shuangshuang Ma, Guohui Fan, Feiya Xu, Xiaomeng Zhang, Yinong Chen, Yuzhi Tao, Yishan Li, Yanshuang Lyu, Peiran Yang, Dingyi Wang, Zhenguo Zhai, Chen Wang

**Affiliations:** 1 Beijing University of Chinese Medicine. National Center for Respiratory Medicine; State Key Laboratory of Respiratory Health and Multimorbidity; National Clinical Research Center for Respiratory Diseases; Institute of Respiratory Medicine, Chinese Academy of Medical Sciences, Department of Pulmonary and Critical Care Medicine, Center of Respiratory Medicine, China-Japan Friendship Hospital, Beijing, China; 2 School of Population Medicine and Public Health, Chinese Academy of Medical Sciences & Peking Union Medical College; State Key Laboratory of Respiratory Health and Multimorbidity; Key Laboratory of Pathogen Infection Prevention and Control (Peking Union Medical College), Ministry of Education, Beijing, China; 3 National Center for Respiratory Medicine; National Clinical Research Center for Respiratory Diseases; Institute of Respiratory Medicine, Chinese Academy of Medical Sciences; Department of Clinical Research and Data Management, Center of Respiratory Medicine, China-Japan Friendship Hospital, Beijing, China; 4 Graduate School of Capital Medical University, Beijing, China; 5 Peking University China‑Japan Friendship School of Clinical Medicine, Beijing, P.R. China; 6 The First Bethune Hospital of Jilin University, Beijing, China; 7 The First Clinical Medical College, Shanxi Medical University, Taiyuan, China; 8 Changping Laboratory, Beijing, China; 9 Chinese Academy of Medical Sciences, Peking Union Medical College, Beijing, China; 10 Department of Physiology, Institute of Basic Medical Sciences, State Key Laboratory of Respiratory Health and Multimorbidity, Chinese Academy of Medical Sciences and School of Basic Medicine, Peking Union Medical College; National Center for Respiratory Medicine; Institute of Respiratory Medicine, Chinese Academy of Medical Sciences, Beijing, China; 11 National Center for Respiratory Medicine; State Key Laboratory of Respiratory Health and Multimorbidity; National Clinical Research Center for Respiratory Diseases; Institute of Respiratory Medicine, Chinese Academy of Medical Sciences, Department of Pulmonary and Critical Care Medicine, Center of Respiratory Medicine, China-Japan Friendship Hospital, Beijing, 100029 China; 12 National Center for Respiratory Medicine; State Key Laboratory of Respiratory Health and Multimorbidity; National Clinical Research Center for Respiratory Diseases, Beijing, 100029 China; 13 Chinese Academy of Medical Sciences, Peking Union Medical College, No 2, East Yinghua Road, Chaoyang District, Beijing, 100029 China; 14 Department of Respiratory Medicine, Capital Medical University, No 2, East Yinghua Road, Chaoyang District, Beijing, 100029 China

**Keywords:** Venous thromboembolism, Renal insufficiency, Efficacy, Safety, Meta-analysis

## Abstract

Patients with venous thromboembolism (VTE) comorbid renal insufficiency (RI) are at higher risk of bleeding and thrombosis. Recommendations in guidelines on anticoagulation therapy for those patients remain ambiguous. The goal of this study is to compare the efficacy and safety between different anticoagulant regimens in VTE patients comorbid RI at different stages of treatment and prophylaxis. We performed English-language searches of Pubmed, EMBASE, and Web of Science (inception to Nov 2022). RCTs evaluated anticoagulants for VTE treatment at the acute phase, extension phase, and prophylaxis in patients with RI and reported efficacy and safety outcomes were selected. The methodological quality of the studies was assessed at the outcome level using the risk-of-bias assessment tool developed by the Cochrane Bias Methods Group. A meta-analysis of twenty-five RCTs was conducted, comprising data from twenty-three articles, encompassing a total of 9,680 participants with RI. In the acute phase, the risk of bleeding was increased with novel oral anticoagulants (NOACs) compared to LMWH (RR 1.29, 95% CI 1.04–1.60). For the prophylaxis of VTE, NOACs were associated with an elevated risk of bleeding compared with placebo (RR 1.31, 95%CI 1.02–1.68). In comparison to non-RI patients, both NOACs and vitamin K antagonists (VKA) could increase the risk of bleeding among RI patients (RR 1.45, 95%CI 1.14–1.84 and RR 1.53, 95%CI 1.25–1.88, respectively) during acute phase, while NOACs may increase the incidence of VTE in RI population (RR 1.74, 95%CI 1.29–2.34). RI patients who are under routine anticoagulation have a significantly higher risk of adverse outcomes. LMWH is the most effective and safe option for VTE treatment or prophylaxis in patients with RI.

**Supplementary Information:**

The online version contains supplementary material available at 10.1186/s12959-023-00576-2.

## Highlights

• Renal insufficient (RI) patients were at significant higher risk of adverse outcomes, especially bleeding, than non-RI patients under the use of routine anticoagulation treatment.

• Low molecular weight heparin (LWMH) would be an optimized option for patients with RI undergoing VTE treatment and prophylaxis, both in terms of efficacy and safety.

• These findings provide comprehensive evidence for the optimal choice of anticoagulants for the treatment and prevention of VTE in patients with comorbid RI.

**Supplementary Information:**

The online version contains supplementary material available at 10.1186/s12959-023-00576-2.

## Introduction

Pulmonary embolism (PE) is a common cardiovascular disease, with an incidence of 60–120 per 100,000 in the United States each year [1]. Chronic kidney disease (CKD) is an independent prognostic factor for the poor prognoses of PE patients including bleeding and death [2].

Anticoagulation is the primary treatment for acute PE, including Vitamin K antagonists (VKAs, e.g. warfarin), unfractionated heparin (UFH), low molecular weight heparin (LMWH), and novel oral anticoagulants (NOACs, e.g. dabigatran, rivaroxaban, apixaban, and edoxaban, etc.), most of which are excreted through kidney [3]. Thus, an individual’s renal function, indicated by estimated glomerular filtration rate (eGFR) or creatinine clearance (CrCl), is vital for determining appropriate anticoagulants. Renal insufficiency could lead to anticoagulant accumulation and abnormal blood concentration escalation. Studies reported that the area under the concentration–time curves (AUCs) and maximum concentrations (Cmax) of dabigatran, rivaroxaban, and apixaban were higher in PE patients with renal insufficiency than those with normal renal function [4–6]. For VKA, a low-dose maintenance treatment is necessary for PE patients with CKD due to its short effective time window and unpredictable pharmacodynamics, even though most metabolic products of warfarin are not excreted through the kidney [7]. Previous studies have suggested that accumulation of anticoagulants in vivo may increase the risk of bleeding [8, 9]. Therefore, selecting the optimal anticoagulant for PE patients with renal insufficiency (RI) to ensure efficacy and safety remains a challenge.

Following the current guidelines, UFH is recommended for PE patients with severe renal insufficiency (CrCl less than 30 mL/min) as initial anticoagulants, followed by VKA, or the dosage of LMWH should be adjusted based on anti-factor-Xa levels [10–13]. However, recommendations on LMWH are still controversial in the European Society of Cardiology/European Respiratory Society (ESC/ERS) guidelines. It is also noteworthy that although NOACs have been widely applied in the general population over the past decades, fewseldom study has been conducted among PE patients with RI. Thus, after thoroughly acquiring data, we aimed to evaluate the efficacy and safety of current anticoagulants to provide additional information from a statistical perspective and identify research orientation for future studies.

## Methods

### Study eligibility and selection

This meta-analysis was conducted according to the PRISMA (Preferred Reporting Items for Systematic Reviews and Meta-Analysis) [14, 15]. We searched Pubmed, Embase, and Web of Science from database inception up to Nov 30, 2022. We used keywords related to venous thromboembolism, kidney function, and anticoagulants in the title and the full text of articles (eTable1 in Supplement 1). We hand-searched reference lists from relevant review articles and meta-analyses to identify any additional studies.

Two reviewers (S.M and F.X) independently performed the review, and disagreements were resolved in a panel discussion with an additional reviewer (G.F). The inclusion criteria for our study were: 1) randomized controlled trials; 2) adult patients (≥ 18 years old) diagnosed with deep vein thrombosis (DVT) and/or PE or required VTE prophylaxis: ((a) eligible patients had to have acute symptomatic proximal DVT, PE or both. (b) eligible patients had to have objectively confirmed, symptomatic proximal deep-vein thrombosis or pulmonary embolism and had been treated for 3 to 12 months with anticoagulation. (c) eligible patients had to have additional risk factors for venous thromboembolism.); 3) treatment with intravenous, subcutaneous or oral anticoagulants (including NOACs, UFH, LMWH, VKA, fondaparinux, etc.) compared with one another or placebo; 4) patients with determined RI (CrCl < 50-60 ml/min or eGFR < 50-60 ml/min/1.73m^2^); 5) reporting efficacy, bleeding outcomes, or both. We excluded observational studies, crossover trials, patients with dialysis-dependent end-stage renal disease (ESRD), and studies published in non-English language and conference abstracts.

### Outcome measures

For treatment evaluation in acute and extension phase, the primary efficacy outcomes were recurrent VTE or death associated with VTE. For prophylaxis, the primary efficacy outcome was the presence of asymptomatic or symptomatic VTE or VTE-related death. The safety outcomes were major bleeding and/or clinically relevant non-major bleeding according to the criteria in the International Society of Thrombosis and Haemostatsis (ISTH) [16].

### Data extraction and quality assessment

Data were extracted independently by two reviewers (F.X and S.M), and disagreements were resolved via consultation with another reviewer (G.F). A standardized form was used to extract the following data: study identifier, study design, location, length of follow-up, number of participants, age, sex, groups of renal function, intervention and control drugs; information relevant to the risk-of-bias assessment (including adherence to and withdrawal from randomized allocation); definition of outcomes and number of events. The methodological quality of each included study was assessed at the outcome level independently by two reviewers (F.X and S.M) using the risk-of-bias assessment tool developed by the Cochrane Bias Methods Group [17] and checked by the third party (G.F) (eFigure1 in Supplement 1).

### Data synthesis and analysis

As outcome data were acquired at different time points, we divided the studies into three categories: prophylactic phases, acute phase, and extension phase. The results were expressed as risk ratios (RRs) with 95% confidence intervals (CIs). A treatment group continuity correction was used if there were 0 events in one group in a trial. Summary estimates were obtained with a random-effects model using the Paule-Mandel method. Statistical heterogeneity across studies was estimated using the I^2^ test, with values of 25%, 50%, and 75% corresponding to low, moderate, and high heterogeneity, respectively. Statistical analyses were performed with Stata, version 16 (Stata, College Station, TX).

## Results

### Search results and characteristics of included studies

**Fig. 1:**
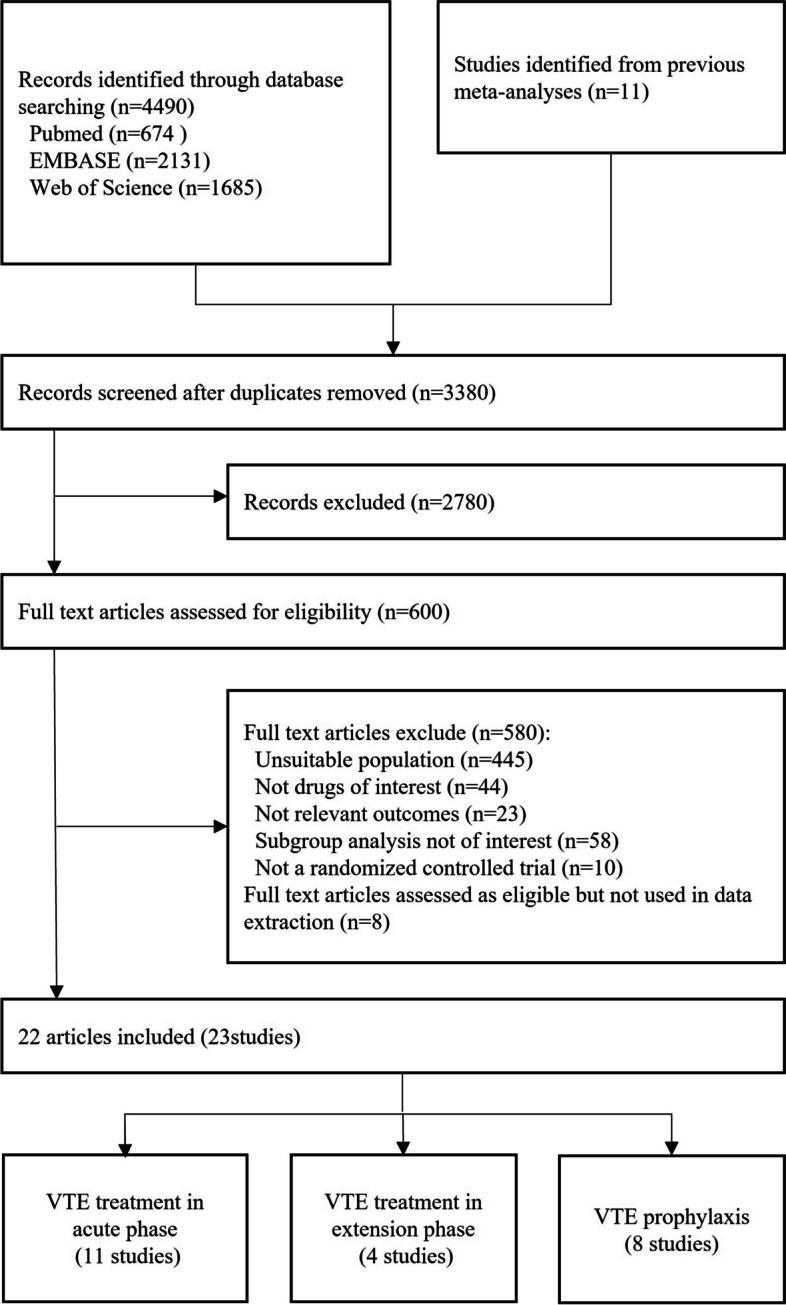
Study flow chart Abbreviations: VTE, venous thromboembolism; RI, renal insufficiency

We obtained 8991 articles through preliminary screening. Duplicate and irrelevant articles were deleted. Six hundred and three articles were evaluated in full text, with 8 articles assessed as eligible but not used in data extraction (*n* = 8). Five hundred and eighty articles were excluded, including inappropriate population (*n* = 445); no drugs of interest (*n* = 44); no relevant outcomes (*n* = 23); subgroup analysis not of interest (*n* = 58); not a randomized controlled trial (*n* = 10). Finally, 23 articles were included for meta-analysis, including data from 25 trials (Fig. 1, Table 1).

**Table 1:** Summary of studies included in meta-analysis

**Study**	**Year**	**Number of patients with RI/ Sample size**	**Renal-function range**	**Study population**	**Intervention/comparison**	**Treatment duration**	**Primary outcome (intervention/ comparison)**	**Bleeding (intervention/ comparison)**
**VTE treatment in acute phase**								
**EINSTEIN-DVT**	2010	250/3429	30 ml/min < CrCl < 50 ml/min	DVT	rivaroxaban/enoxaparin-VKA	3,6,12 m	4/6	13/10
**IRIS**	2011	537/1078	CrCl < 60 ml/min	> 75 year old VTE	tinzaparin/UFH	3 m	8/4	13/17
**EINSTEIN-PE**	2012	404/4817	30 ml/min < CrCl < 50 ml/min	PE	rivaroxaban/enoxaparin-VKA	9 m	7/5	26/34
**Hokusai-VTE**	2013	541/8240	30 ml/min < CrCl < 50 ml/min	VTE	edoxaban/VKA	12 m	8/15	32/32
**AMPLIFY**	2013	327/5395	30 ml/min < CrCl < 50 ml/min	VTE	apixaban/enoxaparin-VKA	6 m	7/7	5/9
**CLOT**	2016	162/676	CrCl < 60 ml/min	cancer associated VTE	dalteparin/VKA	6 m	2/15	15/21
**RE-COVER-I and II**	2017	237/5035	30 ml/min < CrCl < 50 ml/min	VTE	dabigatran/VKA	6 m	0/5	21/29
**CATCH**	2018	131/864	20 ml/min/1.73m^2^ < eGFR < 60 ml/min/1.73m^2^	cancer associated VTE	tinzaparin/VKA	6 m	9/9	13/17
**Hokusai-VTE cancer**	2018	72/1046	30 ml/min < CrCl < 50 ml/min	cancer associated VTE	edoxaban/dalteparin	12 m	2/1	4/1
**AMPLIFY-cancer**	2020	327/1155	25 ml/min < CrCl < 80 ml/min	cancer associated VTE	apixaban/dalteparin	6 m	9/19	10/10
**Caravaggio**	2021	275/1142	30 ml/min < CrCl < 60 ml/min	cancer associated VTE	Apixaban/dalteparin	6 m	3/11	23/13
**Study**	**Year**	**Number of patients with RI/ Sample size**	**Renal-function range**	**Study population**	**Intervention/comparison**	**Treatment duration **	**Primary outcome (intervention/comparison)**	**Bleeding (intervention/comparison)**
**VTE treatment in extension phase**								
**EINSTEIN extention**	2010	86/1188	30 ml/min < CrCl < 50 ml/min	VTE treated 6 to 12 months	rivaroxaban/placebo	6,12 m	1/6	1/2
**RE-MEDY and RE-SONATE**	2013	108/2866	CrCl < 50 ml/min	VTE treated in RECOVER I and II trials	dabigatran/VKA	18 m	0/0	NA
**RE-MEDY and RE-SONATE**	2013	71/1353	CrCl < 50 ml/min	VTE treated in RECOVER I and II trials	dabigatran/placebo	18 m	1/1	NA
**AMPLIFY-EXT**	2013	138/2482	25 ml/min < CrCl < 50 ml/min	VTE treated 6 to 12 months	apixaban/placebo	12 m	5/7	4/2/6
**EINSTEIN CHOICE**	2017	156/3365	30 ml/min < CrCl < 50 ml/min	VTE treated 6 to 12 months	rivaroxaban/aspirin	6,9,12 m	0/3/0	1/4/0
**VTE prophylaxis**								
**CERTIFY**	2011	189/3239	eGFR ≤ 30 ml/min/1.73 m^2^	hospitalised medical patients	certoparin/UFH	9.9 ± 4.3 d	6/2	5/13
**Shorr**	2012	1006/2078	30 ml/min < CrCl < 60 ml/min	THR surgery	desirudin/enoxaparin	8-12 d	24/42	6/2
**Dahl**	2012	159/539	30 ml/min < CrCl < 50 ml/min	joint replacement surgery	dabigatran/enoxaparin	6–10 up to 28-35 d	3/8	0/6
**ADVANCE-2 and 3**	2013	318/6788	30 ml/min < CrCl < 50 ml/min	THR surgery	apixaban/enoxaparin	14d,38 d	1/2	13/11
**APEX**	2016	256/3429	15 ml/min < CrCl < 30 ml/min	acute medical illness	betrixaban/enoxaparin	10 ± 4 d,35-42 d	12/10	3/1
**MARINER**	2018	2183/11962	30 ml/min < CrCl < 50 ml/min	acute medical illness	rivaroxaban/placebo	45 d	18/18	20/10
**CASSINI**	2019	63/841	30 ml/min < CrCl < 50 ml/min	ambulatory cancer patients with a higher risk of VTE	rivaroxaban/placebo	180 d	1/2	NA
**MAGELLAN**	2020	1299/7998	30 ml/min < CrCl < 50 ml/min	≥ 40 years old, acute medical illness	rivaroxaban/enoxaparin	35 d	9/15	36/17
**AVERT**	2022	64/574	30 ml/min < CrCl < 60 ml/min	cancer associated VTE	apixaban/ placebo	180 ± 3 d	0/1	2/3

Renal insufficiency (RI) is usually defined estimated glomerular filtration rate (eGFR) < 60 ml· min-1·1.73 m^−2^ or creatinine clearance (CrCl) < 60 ml/min

*Abbreviations*: *CrCl* creatine clearance, *PE* pulmonary embolism, *RI* renal insufficiency, *THR* total hip replacement, *UFH* unfractionated heparin, *VKA* vitamin K antagonist, *VTE* venous thromboembolism

In the treatment of the acute phase, five trials involving 1759 patients compared the efficacy and safety between NOACs and VKA [18–22]. Two trials compared the efficacy and safety between LMWH and VKA [23, 24]. The Innohep® in Renal Insufficiency Study (IRIS) compared the efficacy and safety between LMWH and UFH [25]. Three trials compared the efficacy and safety between NOACs and LMWH [26–28].

In the treatment of the extension phase, EINSTEIN-CHOICE compared the efficacy and safety of two dosages of NOACs with those of aspirin for up to 1 year after the initial 6 to 12 months of therapy [29]. RE-MEDY and RE-SONATE compared the effectiveness of NOACs vs VKA and NOACs vs placebo [20]. Two trials investigated the efficacy and safety of NOACs vs placebo [30, 31]. Moreover, AMPLIFY-EXT also compared the efficacy and safety of low-dose NOACs vs placebo [30].

For VTE prophylaxis, four trials were included to analyze the safety and efficacy of NOAC and LMWH [32–35]. One trial compared the safety and efficacy between desirudin and LMWH [36]. Three trials investigated the effectiveness of NOACs versus placebo [37–39]. One trial compared the efficacy and safety of LMWH vs UFH [40].

### Comparisons of different anticoagulants among patients with RI

#### VTE treatment in the acute phase

**Fig. 2:**
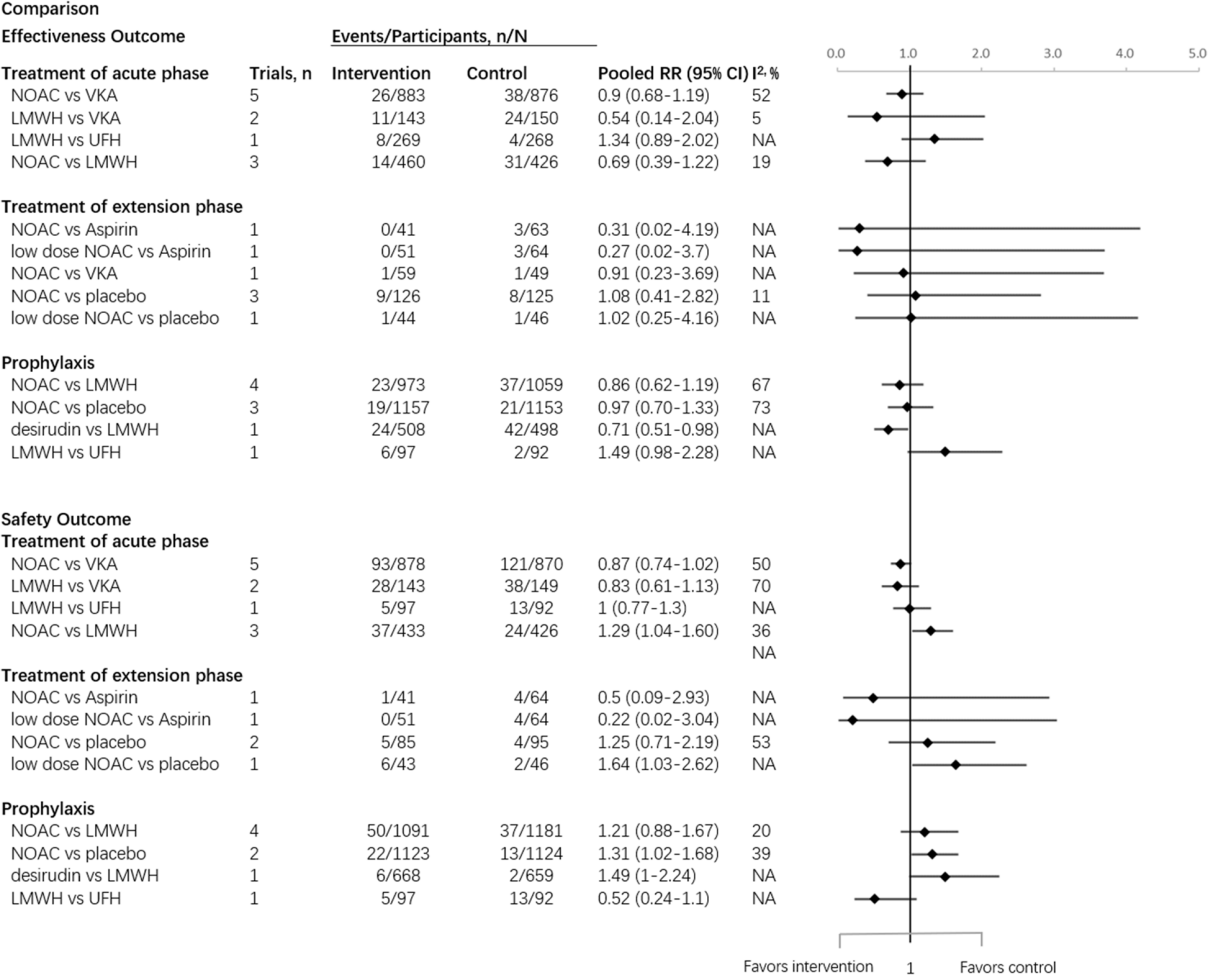
Comparisons of Different Anticoagulants among Patients with RI in Forest Plot Abbreviations: NOAC, novel oral anticoagulants; VKA, vitamin K antagonist; LMWH, low molecular heparin; UFH, unfractionated heparin

In the acute phase, a total of 3,475 VTE patients with RI were involved from eleven studies. Four pairs of comparisons were analyzed: NOACs vs VKA, LMWH vs VKA, LMWH vs UFH and NOACs vs LMWH. Among all the pairs, efficacy endpoints were not significantly different. For safety outcomes, NOACs were associated with an increased risk of bleeding compared to LMWH (RR 1.29, 95%CI 1.04–1.60). No significant difference was observed in the other pairs of comparisons (Fig. 2).

#### VTE treatment in the extension phase

A total of seven studies with 668 VTE patients with RI were enrolled in the extension phase. Five pairs of comparisons were analyzed: NOACs vs aspirin, low-dose NOACs vs aspirin, NOACs vs VKA, NOACs vs placebo, and low-dose NOACs vs placebo. However, no statistically significant difference of efficacy was detected.

For safety outcomes, the placebo was at a lower risk of bleeding than low-dose NOACs (RR 1.64, 95%CI 1.03–2.62), while no significant difference was shown in other comparison pairs (Fig. 2).

#### VTE prophylaxis

The analysis of VTE prophylactic phases contained nine studies involving 5,537 VTE patients with RI. Four pairs of comparisons were analyzed: NOACs vs aspirin, low-dose NOACs vs aspirin, NOACs vs VKA, NOACs vs placebo and low-dose NOACs vs placebo. Compared with LMWH, desirudin was associated with a lower risk of VTE occurrence (RR 0.71, 95%CI 0.51–0.98) but a higher risk of bleeding (RR 1.49, 95%CI 1.00–2.24) Meanwhile, NOACs significantly increased the risk of bleeding compared with placebo (RR 1.31, 95%CI 1.02–1.68) (Fig. 2).

### Efficacy and safety of different anticoagulants among patients with and without RI

We also conducted meta-analyses on participants with and without RI who had been prescribed each anticoagulant, to investigate the efficacy and safety of one particular anticoagulant in both populations.

#### VTE treatment in the acute phase

**Fig. 3:**
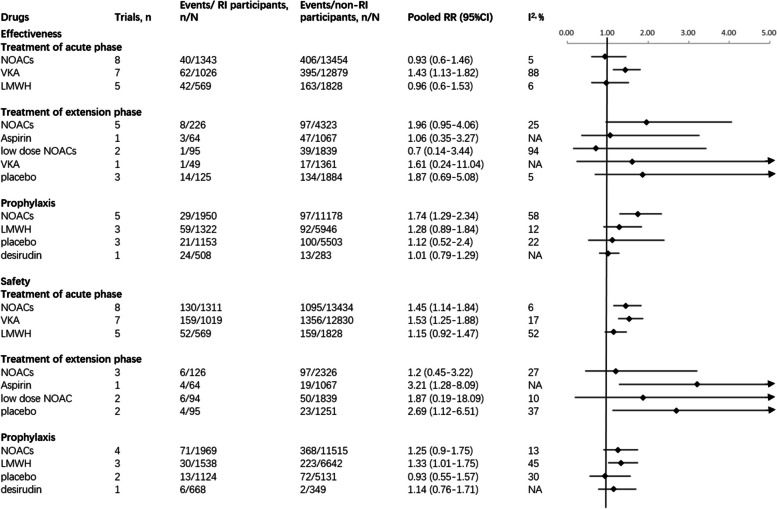
Effects of Different Anticoagulants among Patients with and without RI in Forest Plot Abbreviations: NOAC, novel oral anticoagulants; VKA, vitamin K antagonist; LMWH, low molecular heparin.

In the acute phase of the treatment studies, a total of 2,938 VTE patients with RI and 28,161 VTE patients without RI were included. Three anticoagulants were analyzed: NOACs, VKA, and LMWH. Among the seven studies using VKA, the risk of death or recurrent VTE was significantly higher in the RI patients (RR 1.43, 95%CI 1.13–1.82). For safety outcomes, NOACs and VKA might lead to a higher risk of bleeding among the RI patients compared with those without RI (RR 1.45, 95%CI 1.14–1.84 and RR 1.53, 95%CI 1.25–1.88, respectively) (Fig. 3).

#### VTE treatment in the extension phase

In the extention phase of the treatment studies, a total of 559 VTE patients with RI and 10,474 VTE patients without RI were included. Five anticoagulants were analyzed: NOACs, aspirin, low-dose NOACs, VKA and placebo. The efficacy of these drugs between RI and non-RI patients was found to be similar. Aspirin may increase the risk of bleeding in RI patients (RR 3.21, 95%CI 1.28–8.09) (Fig. 3).

#### VTE prophylaxis

In the VTE prophylaxis studies, a total of 4,933 VTE patients with RI and 22,910 VTE patients without RI were included. Four anticoagulants were analyzed: NOACs, LMWH, desirudin and placebo. Compared with the non-RI population, NOACs may increase the occurrence of VTE in RI population (RR 1.74, 95%CI 1.29–2.34). There was no difference among groups treated with NOACs, placebo and desirudin, but LMWH could increase the risk of bleeding in the RI population (RR 1.33, 95%CI 1.01–1.75) (Fig. 3).

## Discussion

**Fig. 4:**
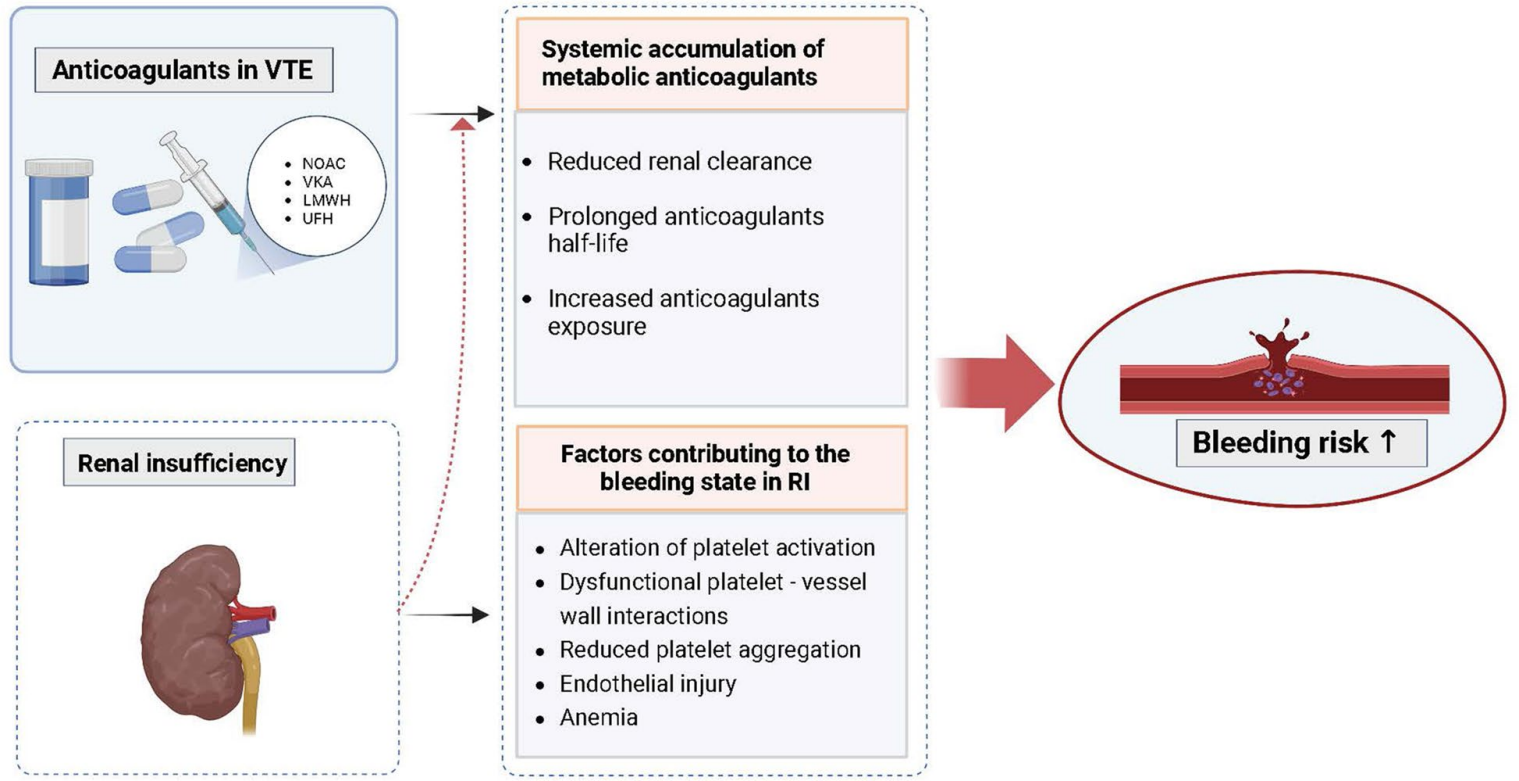
Mechanisms of increased bleeding risk in VTE patients with RI Abbreviations: VTE, venous thromboembolism; RI, renal insufficiency; NOAC, novel oral anticoagulant; VKA, vitamin K antagonist; LMWH, low molecular heparin

In this study, we updated recent studies and innovatively divided them into acute-extension-prophylaxis stages as pathophysiological status differed. The results of our study showed that NOAC may increase the risk of bleeding in RI population, compared with non-RI patients, under the use of routine anticoagulation treatment. So far, this study is the most comprehensive study on the efficacy and safety of anticoagulant for treatment and prophylaxis of VTE patients with RI (Fig. 4).

The choice of anticoagulant remains controversial for VTE patients comorbid RI, who are at high risk of both bleeding and thrombosis. Clinical evidence was insufficient on the advantages or disadvantages of different treatment strategies. Ha et al. conducted a thorough meta-analysis of anticoagulants among patients with CKD in 2019 [41] but did not recommend a single anticoagulant regimen of the best efficacy-safety balance for VTE patients. The results of our previous study, a network meta-analysis of anticoagulants among VTE patients with RI, provided clues on the anticoagulation regimens for patients under such conditions [42]. In this study, we reviewed previous studies and further determined the risk and efficacy of specific anticoagulants in detail, which is helpful in further recommendations of anticoagulants.

The pharmacokinetics of anticoagulants in VTE patients with RI are characterized as reduced renal clearance, prolonged half-life and increased exposure to anticoagulant drugs thus elevated the risk of bleeding. Notably, one of the most wildly applied anticoagulants, LMWH, is preferentially excreted through the kidneys, so the administration should be cautious in patients with RI to reduce the drug accumulation and minimize the risk of bleeding. Therefore, guidelines recommend an adjusted dose of LMWH among patients with RI and monitor the level of anti-Xa [43–45]. In this way, the adjusted dose of LWWH appears to be safer in the acute PE population with RI, even for those with severe RI or other diseases co-administered with various drugs [46, 47]. Besides, different species of LMWHs have particular molecular weights and bioactivities, for example, studies found that tinzaparin (LMWH above approximately 5,000 Da) was less likely to accumulate than enoxaparin (LMWH below approximately 5,000 Da) in subjects with RI [48, 49]. Therefore, LMWH with lower dependence on renal clearance was preferred in clinical practice.

NOACs not only reduce the frequency of treatment monitoring compared with VKA, but also have similar efficacy in reducing the risk of VTE and a lower risk of overall bleeding (especially intracranial bleeding) in the general population [19]. Harel et al. demonstrated that among patients with CKD (defined as a CrCl ≤ 50 ml/min), no significant difference in efficacy existed between NOACs and VKA [50]. Siontis et al. reported that no difference was found in the rate of thromboembolic events between NOACs and VKA, with a lower risk of bleeding in patients taking NOACs in patients with end-stage kidney disease and atrial fibrillation [51]. Our study indicated that NOACs were comparable with VKA on efficacy, but superior to VKA on safety. However, NOACs were related to a higher risk of bleeding than LMWH during the acute phase. Furthermore, given the differences in renal-dependent excretion, the risks of bleeding among different NOACs vary. For example, dabigatran is mainly excreted through the kidneys thus more likely to accumulate in VTE patients with RI. Previous studies have found severe bleeding events with dabigatran in patients with RI [52, 53]. In general, LWMH would be an optimized option both in efficacy and safety for patients with RI, for VTE treatment or prophylaxis.

Additionally, we innovatively conducted the comparisons of efficacy and safety among different anticoagulants between patients with and without RI. Adverse outcomes occurred more frequent among patients with RI when applied with VKA in the acute phase or with NOACs in prophylaxis, and LMWH for VTE patients with RI might be as effective as those without RI. For safety consideration, patients with RI had higher risk of bleeding when applied with whichever anticoagulants, due to the pathophysiological changes in patients with RI. However, because of the limited number of RI population, there might be inevitable bias in comparison. More clinical studies particularly among the RI population are further required.

Potential limitations remain in our study: firstly, most of the clinical trials excluded patients with CrCl < 30 mL/min. The lack of evidence-based guidelines strongly suggests that RCTs are required to address the unmet need in this population, replenishing new and strong evidences. According to current guidelines, patients with severe renal insufficiency were recommended UFH [44], but the problems on accessibility and convenience remained. In real-world settings, most patients with CrCl < 30 mL/min were prescribed LMWH [46], thus, more investigations of pharmacokinetics were of great importance for those patients. Second, CrCl was estimated before enrollment of VTE patients and its dynamic changes were not monitored during treatment, which might impact the outcome. In addition, different compounds of LMWH and NOACs which were applied and comorbidities in different studies may also impact the prognoses of participators.

## Conclusion

RI patients were at significantly higher risk than non-RI patients under the use of routine anticoagulation treatment. LWMH might be an optimized option both on efficacy and safety for patients with RI, for VTE treatment or prophylaxis.

### Supplementary Information

**Additional file 1: eTable 1.** Search strategies. **eFigure 1. **Classification of risk of bias for each study domain among VTE patients with based on Cochrane tool.

## Data Availability

Not applicable.
