# Supplementary material for: Efficacy and safety of anticoagulant for treatment and prophylaxis of VTE patients with renal insufficiency: a systemic review and meta-analysis
Source: Thromb J. 2024 Feb 5;22:17. doi: 10.1186/s12959-023-00576-2 (PMC10840151; doi:10.1186/s12959-023-00576-2)
Supplement: Supplementary file 1 — Additional file 1: eTable 1. Search strategies. eFigure 1. Classification of risk of bias for each study domain among VTE patients with based on Cochrane tool. [file 12959_2023_576_MOESM1_ESM.docx]

**Supplementary materials**

eTable 1. **search strategies**

eFigure 1. **Classification of risk of bias for each study domain among VTE patients with based on Cochrane tool.**

eTable 1 **Full search strategies for published studies**

| **Pubmed** |
| --- |
| (anticoagulant*[Title/Abstract] OR anticoagulation*[Title/Abstract] OR LMWH[Title/Abstract] OR VKA[Title/Abstract] OR NOAC*[Title/Abstract] OR DOAC*[Title/Abstract] OR UFH[Title/Abstract] OR low molecular weight heparin[Title/Abstract] OR unfractionated heparin[Title/Abstract] OR warfarin[Title/Abstract] OR direct oral anticoagulant*[Title/Abstract] OR novel[Title/Abstract] OR anticoagulant*[Title/Abstract] OR dabigatran[Title/Abstract] OR rivaroxaban[Title/Abstract] OR apixaban[Title/Abstract] OR edoxaban[Title/Abstract] OR enoxaparin[Title/Abstract] OR dalteparin[Title/Abstract] OR tinzaparin[Title/Abstract] OR fondaparinux[Title/Abstract] OR argatroban[Title/Abstract])  AND  (venous thromboembo*[Title/Abstract] OR pulmonary embo*[Title/Abstract] OR pulmonary thromboembo*[Title/Abstract] OR VTE[Title/Abstract] OR PE[Title/Abstract] OR PTE[Title/Abstract])  AND  (chronic kidney disease[Title/Abstract] OR renal failure[Title/Abstract] OR renal impairment[Title/Abstract] OR end stage renal disease[Title/Abstract] OR end-stage renal disease[Title/Abstract] OR dialysis[Title/Abstract] OR kidney failure[Title/Abstract] OR kidney insufficiency[Title/Abstract] OR kidney dysfunction[Title/Abstract] OR renal insufficiency[Title/Abstract] OR renal dysfunction[Title/Abstract] OR CKD[Title/Abstract] OR ESRD[Title/Abstract] OR ESKD[Title/Abstract]) |

eTable 1 **Full search strategies for published studies（Continued）**

| **EMBASE** |
| --- |
| ((anticoagulant*):ab,ti OR (anticoagulation*):ab,ti OR (LMWH):ab,ti OR (VKA):ab,ti OR (NOAC*):ab,ti OR (DOAC*):ab,ti OR (UFH):ab,ti OR (low molecular weight heparin):ab,ti OR (unfractionated heparin):ab,ti OR (warfarin):ab,ti OR (direct oral anticoagulant*):ab,ti OR (novel):ab,ti OR (anticoagulant*):ab,ti OR (dabigatran):ab,ti OR (rivaroxaban):ab,ti OR (apixaban):ab,ti OR (edoxaban):ab,ti OR (enoxaparin):ab,ti OR (dalteparin):ab,ti OR (tinzaparin):ab,ti OR (fondaparinux):ab,ti OR (argatroban):ab,ti )  AND  ((venous thromboembo*):ab,ti OR (pulmonary embo*):ab,ti OR (pulmonary thromboembo*):ab,ti OR (VTE):ab,ti OR (PE):ab,ti OR (PTE):ab,ti )  AND  ((chronic kidney disease):ab,ti OR (renal failure):ab,ti OR (renal impairment):ab,ti OR (end stage renal disease):ab,ti OR (end-stage renal disease):ab,ti OR (dialysis):ab,ti OR (kidney failure):ab,ti OR (kidney insufficiency):ab,ti OR (kidney dysfunction):ab,ti OR (renal insufficiency):ab,ti OR (renal dysfunction):ab,ti OR (CKD):ab,ti OR (ESRD):ab,ti OR (ESKD):ab,ti ) |
| **Web of Science** |
| TS=(anticoagulant* OR anticoagulation* OR LMWH OR VKA OR NOAC* OR DOAC* OR UFH OR low molecular weight heparin OR unfractionated heparin OR warfarin OR direct oral anticoagulant* OR novel OR anticoagulant* OR dabigatran OR rivaroxaban OR apixaban OR edoxaban OR enoxaparin OR dalteparin OR tinzaparin OR fondaparinux OR argatroban)  AND  TS= (venous thromboembo* OR pulmonary embo* OR pulmonary thromboembo* OR VTE OR PE OR PTE)  AND  TS= (chronic kidney disease OR renal failure OR renal impairment OR end stage renal disease OR end-stage renal disease OR dialysis OR kidney failure OR kidney insufficiency OR kidney dysfunction OR renal insufficiency OR renal dysfunction OR CKD OR ESRD OR ESKD) |


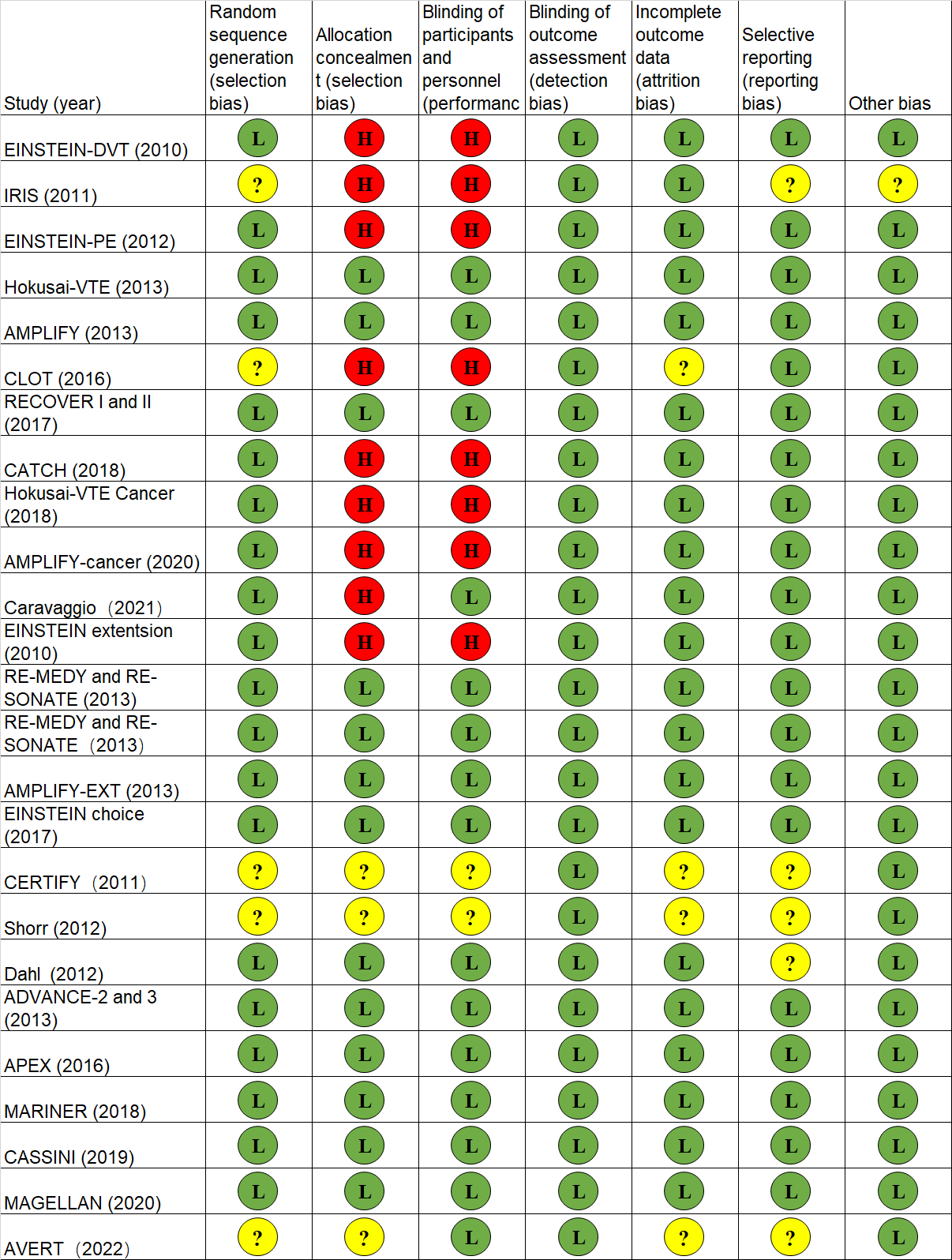


eFigure 1. Classification of risk of bias for each study domain among VTE patients with based on Cochrane tool.

Abbreviations: H, high risk; ?, unclear risk; L, low risk
